# Receptor-targeted nanoparticles modulate cannabinoid anticancer activity through delayed cell internalization

**DOI:** 10.1038/s41598-022-05301-z

**Published:** 2022-01-25

**Authors:** Matilde Durán-Lobato, Josefa Álvarez-Fuentes, Mercedes Fernández-Arévalo, Lucía Martín-Banderas

**Affiliations:** Dpto. Farmacia y Tecnología Farmacéutica, Facultad de Farmacia, Universidad de Sevilla, C/Prof. García González n °2, 41012 Seville, Spain

**Keywords:** Nanoscience and technology, Drug delivery, Nanotechnology in cancer

## Abstract

Δ^9^-tetrahydrocannabinol (Δ^9^-THC) is known for its antitumor activity and palliative effects. However, its unfavorable physicochemical and biopharmaceutical properties, including low bioavailability, psychotropic side effects and resistance mechanisms associated to dosing make mandatory the development of successful drug delivery systems. In this work, transferring (Tf) surface-modified Δ^9-^THC-loaded poly(lactide-co-glycolic) nanoparticles (Tf-THC-PLGA NPs) were proposed and evaluated as novel THC-based anticancer therapy. Furthermore, in order to assess the interaction of both the nanocarrier and the loaded drug with cancer cells, a double-fluorescent strategy was applied, including the chemical conjugation of a dye to the nanoparticle polymer along with the encapsulation of either a lipophilic or a hydrophilic dye. Tf-THC PLGA NPs exerted a cell viability decreased down to 17% vs. 88% of plain nanoparticles, while their internalization was significantly slower than plain nanoparticles. Uptake studies in the presence of inhibitors indicated that the nanoparticles were internalized through cholesterol-associated and clathrin-mediated mechanisms. Overall, Tf-modification of PLGA NPs showed to be a highly promising approach for Δ^9^-THC-based antitumor therapies, potentially maximizing the amount of drug released in a sustained manner at the surface of cells bearing cannabinoid receptors.

## Introduction

The potential therapeutic applications of marijuana, firstly reported in 1997 by the National Institutes of Health (NIH, USA), are attributed to a great extent to its main component, Δ^9^-tetrahydrocannabinol (Δ^9^-THC)^1^. This cannabinoid continues to attract special attention in oncology due to its palliative effects and antitumor activity; Δ^9^-THC has been reported to inhibit tumor angiogenesis and cell growth in malignant tissues, leading to cell death^2–4^. Unfortunately, this drug presents a major challenge for the design of a suitable pharmaceutical dosage for clinical use, due to its unfavorable physicochemical properties; high instability, oily-resin nature, low solubility in water and low bioavailability, along with psychotropic side effects^5^. In addition, its anticancer effect is frequently subject to resistance mechanisms, some of them strongly related to the actual dose reaching the target site^4^, which is subsequently dependent on the dosage form. Since classical pharmaceutical delivery systems such as oral aerosols, transdermal patches and suppositories have not achieved satisfactory results so far, strategies based on micro- and nanotechnology were proposed, including liposomes^6^, micelles^7^ and self-emulsifying drug delivery systems (SEDDS)^8,9^. Our research team proposed for the first time cannabinoid-loaded PLGA nanoparticles (PLGA NPs)^10^, and also introduced Δ^9^-THC-loaded PLGA nanoparticles (THC-PLGA NPs) with further surface modifications to modulate the behavior of the nanocarriers^11^. The presented results, along with the above-discussed challenging aspects of Δ^9^-THC anticancer therapy, highlighted the potential benefits that could be achieved by incorporating a targeting ligand in the formulation^12^, yet unexplored in Δ^9^-THC formulations.

In this work, several steps forward were taken by including a targeting ligand and a multifunctional fluorescent labeling strategy in the presented approach. Transferrin (Tf), a targeting moiety for cancer cells based on a higher expression of the Tf receptor in tumor cells^13–16^, was coupled to Δ^9^-THC-loded PLGA NPs to modulate the interaction of the particles with the target cells. In addition, a double fluorescent labeling of the formulations, both through chemical linkage to the polymer and through dye encapsulation, was performed in order to selectively track the internalization pathway and intracellular fate of both the carrier and the cargo. In addition, both a hydrophobic and a hydrophilic dye were encapsulated to enable a closer comparison to published literature employing different labeling approaches. The resulting formulations were evaluated in order to correlate the modulation of their anticancer effect with their cell internalization mechanics.

## Results

### Characterization of NPs

Results of average size, size distribution, ZP and Tf CE (%) of all formulations are collected in Table 1. All the formulations presented a nanometer size (260–332 nm) with narrow size distributions (PDI 0.106–0.219), with no statistically significant differences between the formulations assayed (p < 0.05). Regarding ZP values, a slight decrease could be observed when FITC-PLGA was employed to produce NPs instead of plain PLGA (from − 30 ± 5 mV in PLGA NPs to − 18 ± 4 mV in FITC-PLGA NPs), as well as in Tf-modified plain PLGA formulations (from − 30 ± 5 mV in PLGA NPs to − 21 ± 2 mV in the case of Tf-PLGA NPs and − 20 ± 3 mV in the case of Tf-THC-PLGA NPs). In the case of Tf-modified FITC-PLGA NPs, the decreased in ZP values was slightly accentuated with regard to the rest of formulations (− 15 ± 2 mV). The loading of the drug and fluorophores did not induce statistically significant changes in the ZP of formulations with regard to the corresponding blank formulation (− 30 ± 5 mV in PLGA NPs and − 35 ± 5 mV in THC-PLGA NPs; − 21 ± 2 mV in Tf-PLGA NPs and − 20 ± 3 mV in Tf-THC-PLGA NPs; − 18 ± 4 mV in FITC-PLGA NPs and − 20 ± 2 mV and − 19 ± 4 mV in NR- and Rh-FITC-PLGA NPs, respectively; − 15 ± 2 mV in Tf-FITC-PLGA NPs and − 14 ± 1 mV and − 15 ± 2 mV in Tf-NR- and Tf-Rh-FITC-PLGA NPs, respectively) (Table 1).

**Table 1:** Physicochemical properties of the formulations.

Formulation	Size (nm)	PDI	ZP (mV)	Tf CE (%)	Tf μg/mg NPs
PLGA NPs	280 ± 35	0.146	− 30 ± 5	–	–
Tf-PLGA NPs	290 ± 30	0.201	− 21 ± 2*	79 ± 5	7.8 ± 0.5
THC-PLGA NPs	332 ± 47	0.219	− 35 ± 5	–	–
Tf-THC-PLGA NPs	284 ± 40	0.105	− 20 ± 3**	75 ± 6	7.4 ± 0.6
FITC-PLGA NPs	316 ± 38	0.179	− 18 ± 4**	–	–
Tf-FITC-PLGA NPs	260 ± 35	0.119	− 15 ± 2***	61 ± 6	6.1 ± 0.6
NR-FITC-PLGA NPs	288 ± 30	0.209	− 20 ± 2**	–	–
Tf-NR-FITC-PLGA NPs	289 ± 19	0.106	− 14 ± 1****	65 ± 4	6.4 ± 0.4
Rh-FITC-PLGA NPs	282 ± 32	0.153	− 19 ± 4**	–	–
Tf- Rh-FITC-PLGA NPs	302 ± 38	0.113	− 15 ± 2***	51 ± 5	5.1 ± 0.5

*Tf CE* Tf conjugation efficacy. Statistically significant differences in ZP values are referred to plain PLGA NPs as control group; *p < 0.0332; **p < 0.0021; ***p < 0.0002; ****p < 0.0001.

**Figure 1:**
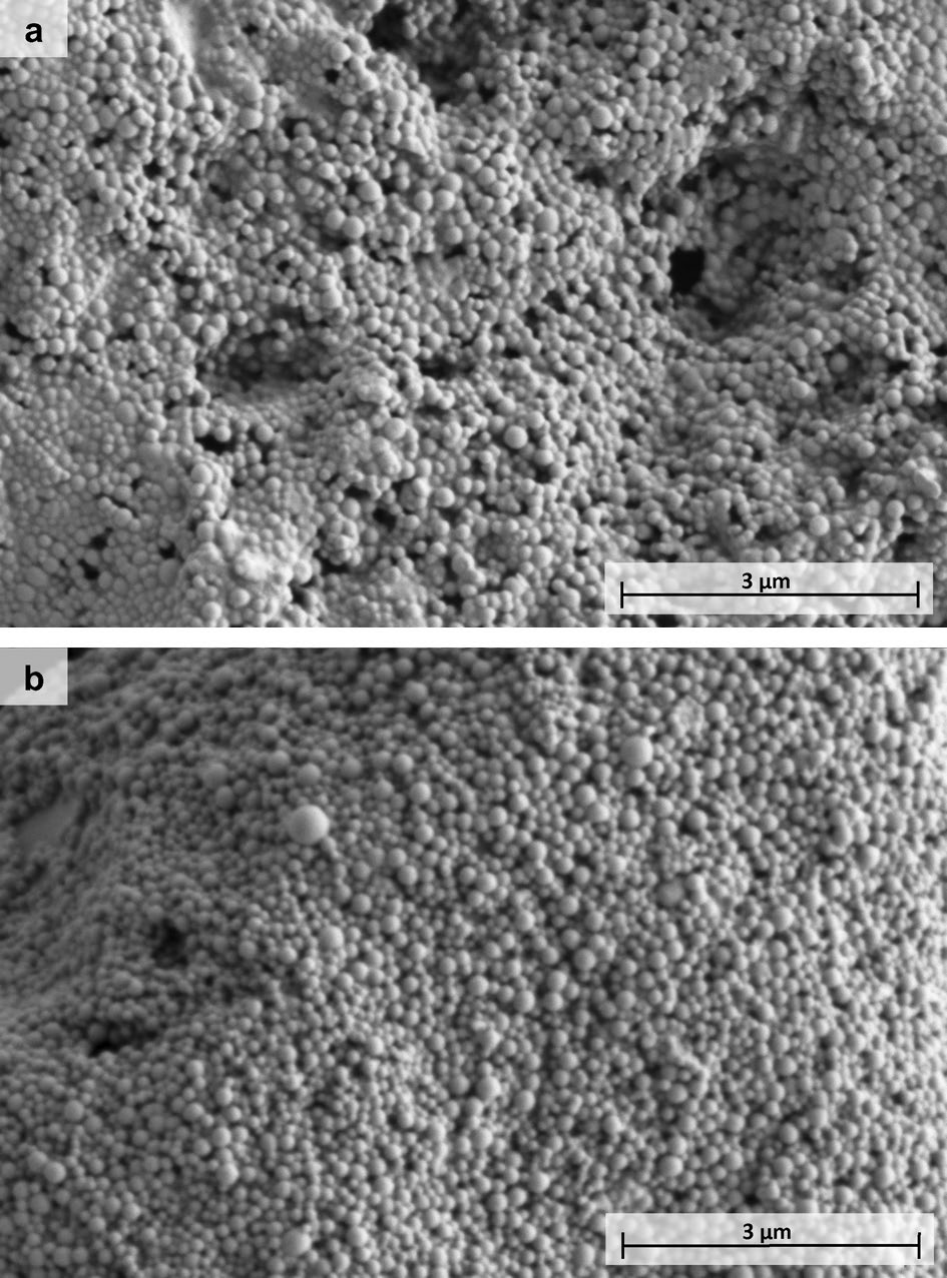
SEM images of (**a**) THC-PLGA-NPs and (**b**) Tf-THC-PLGA NPs.

SEM imaging (Fig. 1) of the formulations presented monodispersed populations of spherical and symmetrical nanoparticles with size in accordance with DLS characterization (Table 1), with no visible difference between Tf-modified and plain NPs.

EE of Δ^9^-THC was 91.1 ± 1.0% in THC-PLGA NPs and 90.9 ± 3.5% in Tf-THC-PLGA NPs, determining a DL of 4.55 ± 0.1%, and 4.54 ± 0.4%, respectively. The quantification of unreacted FITC established the conjugation of FITC at 12.90 μg/mg of NPs, which translates into a 1.29% of CE. Regarding Tf CE, high values were obtained after coupling to plain PLGA NPs (75–79%), with lower values in the case of FITC-PLGA formulations (61–65%), being the lowest CE value the one obtained with Tf-Rh-FITC-PLGA NPs (51%). The mass ratio of Tf to NPs corresponding to the CE% values is displayed in Table 1.

### In vitro drug release

**Figure 2:**
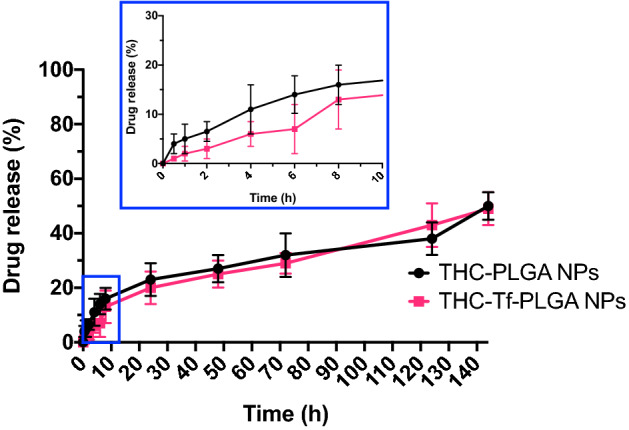
Δ9-THC (%) released from THC-PLGA NPs and Tf-THC-PLGA NPs.

Plain THC-PLGA-NPs and Tf-THC-PLGA NPs presented an extended release profile over several days, with a faster release phase in the first 10 h of the study, followed by a slower phase attaining up to 50% release of the total drug content in the period assayed (140 h) (Fig. 2). No statistically significant differences were observed between plain and Tf-modified Δ^9^-THC-loaded NPs.

### Cellular toxicity

**Figure 3:**
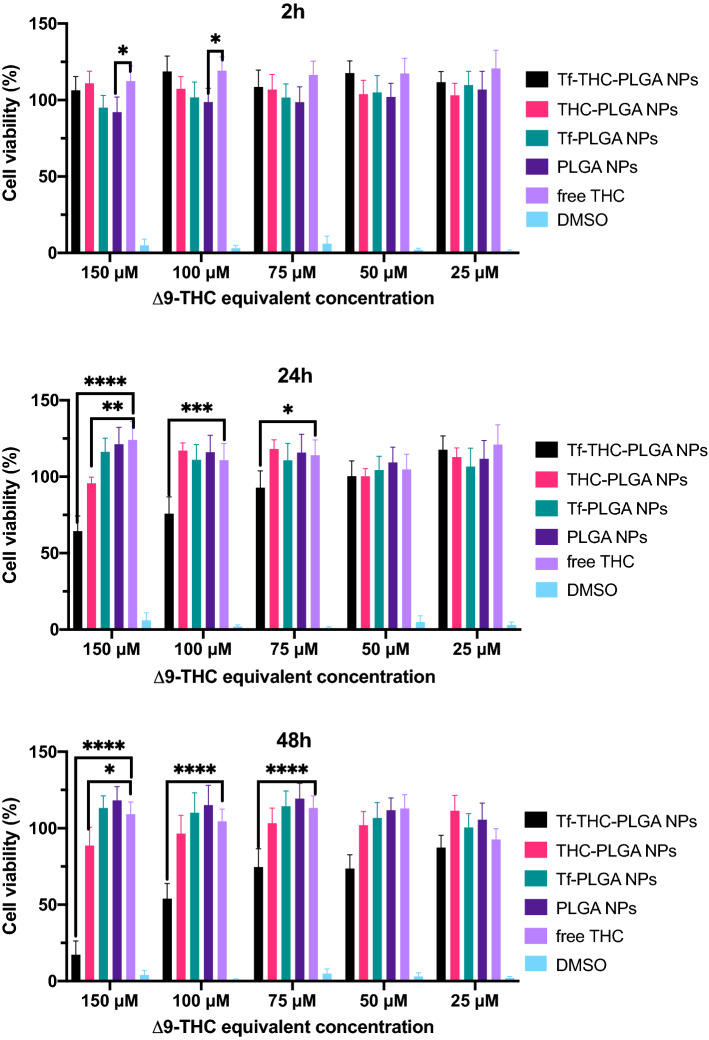
MTT data obtained after 2 h, 24 h and 48 h of incubation. Statistically significant differences in cell viability (%) values are referred to plain PLGA NPs as control group; *p < 0.0332; **p < 0.0021; ***p < 0.0002; ****p < 0.0001.

The results from MTT assays performed at 2, 24 and 48 h of incubation are displayed in Fig. 3. Near 0% viability values obtained with DMSO validated the assay, and both PLGA NPs and Tf-PLGA NPs proved absence of toxicity during the time points assayed. Free THC led to higher values of viability near 120% in the first 2 h, which stabilized around 100% at 24 and 48 h.

In the case of THC-PLGA NPs, similar values were obtained with regard to blank PLGA-NPs and Tf-PLGA NPs at all-time points and concentrations assayed, with the exception of the highest concentration (150 μM equivalent dose of Δ^9^-THC, 0.96 mg/mL of THC-PLGA NPs) at 24 h (95% viability) and 48 h (89% viability). Interestingly, Tf-modified THC-loaded NPs (Tf-THC-PLGA NPs) presented a completely different behaviour; at 2 h, values near or slightly higher than 100% viability were obtained, while a concentration-dependent decrease in viability was observed at 24 h at 150 μM, 100 μM and 75 μM equivalent dose (64, 75 and 92% cell viability respectively) that was markedly noticeable at 48 h at all concentrations assayed (cell viability values in the range 17–87%), attaining 17% cell viability at the highest concentration (150 μM equivalent dose, 0.96 mg/mL).

### Flow cytometry analysis of NPs uptake: energy dependence and inhibition

**Figure 4:**
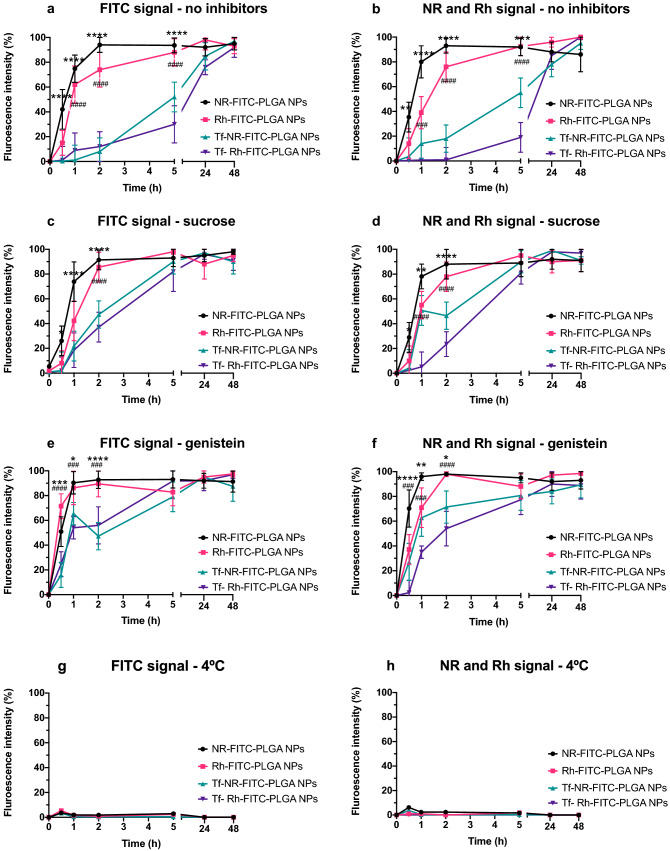
Flow cytometry analysis of NPs uptake under the different conditions assayed. (**a**,**c**,**e**,**g**) present the fluorescence intensity collected in the green channel under the different conditions assayed: (**a**) no uptake inhibition; (**c**) clathrin-dependent endocytosis inhibition (sucrose); (**e**) caveolae-dependent endocytosis inhibition (genistein); and (**g**) energy-dependent uptake inhibition (4 °C) respectively, representing the fluorescence intensity due to FITC associated to the polymer in NPs, and thus associated to the presence of NPs. (**b**,**d**,**f**,**h**) show the corresponding fluorescence intensity obtained in the red channel for the lipophilic fluorophore (NR) and the hydrophilic fluorophore (Rh), associated to the uptake of either lipophilic or hydrophilic molecules encapsulated in the NPs under the different conditions of uptake inhibition assayed. Statistically significant differences in fluorescence intensity (%) values between plain NR-FITC-PLGA NPs and Tf- NR-FITC-PLGA NPs are highlighted as *p < 0.0332; **p < 0.0021; ***p < 0.0002; ****p < 0.0001. Statistically significant differences in fluorescence intensity (%) values between plain Rh-FITC-PLGA NPs and Tf- Rh-FITC-PLGA NPs are highlighted as ^#^p < 0.0332; ^##^p < 0.0021; ^###^p < 0.0002; ^####^p < 0.0001.

The results obtained from the analysis of NPs uptake and their cargo is depicted in Fig. 4. Figure 4a,c,e,g present the fluorescence intensity collected in the green channel under the different conditions assayed: (a) no uptake inhibition; (c) clathrin-dependent endocytosis inhibition (sucrose); (e) caveolae-dependent endocytosis inhibition (genistein); and (g) energy-dependent uptake inhibition (4 °C) respectively, representing the fluorescence intensity due to FITC associated to the polymer in NPs, and thus associated to the presence of NPs.

Figure 4b,d,f,h show the corresponding fluorescence intensity obtained in the red channel for the lipophilic fluorophore (NR) and the hydrophilic fluorophore (Rh), associated to the uptake of either lipophilic or hydrophilic molecules encapsulated in the NPs under the different conditions of uptake inhibition assayed.

Figure 4a shows a similar uptake profile of NR- and Rh- FITC-PLGA NPs, with an initial exponential phase that plateaus from 2 h to the end of the study. In contrast, Tf-NR- and Tf-Rh-FITC-PLGA NPs presented an initial lapse of fluorescence intensity in the first 2 h with regard to plain FITC-PLGA NPs, when the uptake increased thereafter.

The corresponding results of fluorescence intensity from the encapsulated fluorophores (Fig. 4b) showed an overall similar profile to the nanoparticle uptake profile (FITC signal, Fig. 4a), albeit with differences in the values obtained for Tf-modified NPs loaded with either NR or Rh.

When clathrin-dependent mechanisms were inhibited by sucrose (Fig. 4c,d), the uptake of Tf-modified FITC-PLGA NPs was increased at the first time points of incubation (0.5 to 2 h) when compared to standard conditions (no inhibitors, Fig. 4a,b), although still maintained a slower uptake profile than their plain NPs counterparts. The values obtained from the tracking of encapsulated dyes (Fig. 4d) also showed the same trend as their corresponding nanoparticle signal (Fig. 4c), with slight differences between NR- and Rh- signal from Tf-modified NPs.

In addition, when genistein was employed to inhibit caveolae-dependent uptake processes (Fig. 4e,f), a similar effect could be observed, where Tf-modified formulations presented a higher uptake rate, yet lower than plain nanoparticles, at the first incubation times compared to control conditions (Fig. 4a,b). Once again, a similar trend could be observed both from the nanoparticles signals (Fig. 4e) and the encapsulated dyes signals (Fig. 4f), with a higher variation between the values obtained with NR and Rh for the same nanoparticle type.

Finally, Fig. 4g,h present fluorescence intensity values near 0% for both NPs and the encapsulated molecules corresponding to cell cultures incubated at 4 °C to inhibit energy-dependent mechanisms.

### Intracellular tracking of NPs and their cargo

**Figure 5:**
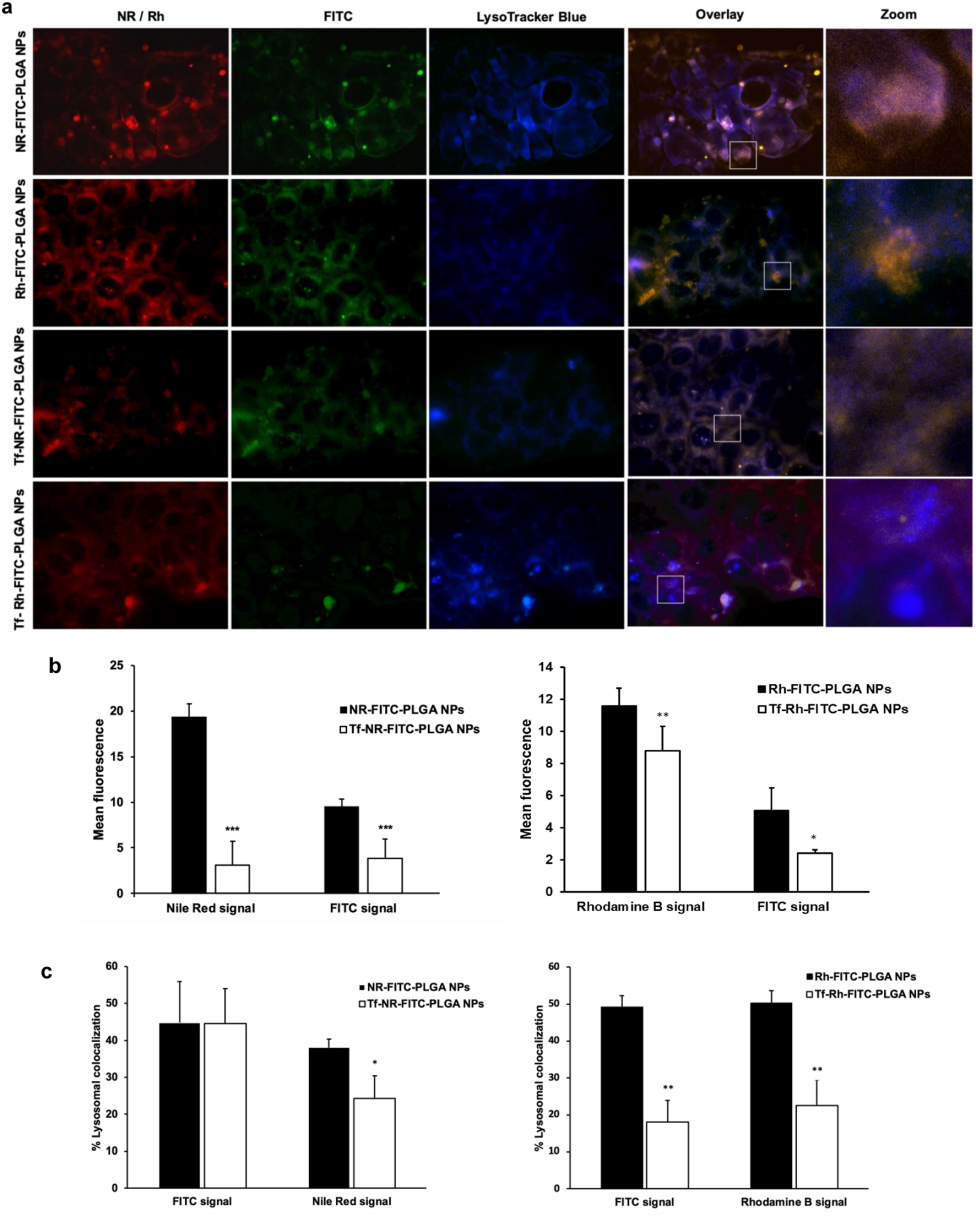
Intracellular trafficking analysis of the formulations upon interaction with the cells. (**a**) Fluorescent microscopy images of Caco-2 cells incubated with NR- and Rh- loaded, FITC-conjugated, plain and Tf-modified NPs for 2 h. Red represents the fluorescence of the loaded fluorophore (i.e. Nile Red or Rhodamine B); green represents the conjugated fluorophore FITC; blue represents lysosomes stained with LysoTracker Blue. The insets highlight a magnified view of the marked areas, showing colocalization of the NPs with lysosomes. (**b**) Percent fluorescence intensity obtained in the images from each fluorophore from plain and Tf-modified formulations. (**c**) Lysosomal percent colocalization. The colocalization analysis was performed with default M1 and M2 coefficients in ImageJ software. Statistically significant differences are highlighted as *p < 0.05; **p < 0.01; ***p < 0.001.

Figure 5 displays the fluorescent microscopy images resulting from the interaction of dual-fluorescently-labelled NPs with Caco-2 cells. Strong fluorescent signals from both FITC and encapsulated dyes confirmed the interaction of the formulations with the cells (Fig. 5a). Overall, higher fluorescence intensity was obtained in cells incubated with plain formulations vs. Tf-modified formulations (Fig. 5a,b), suggesting a higher degree of uptake for plain nanoparticles, in agreement with flow cytometry results. In addition, the overlapping of fluorescence signals from the chemically conjugated and encapsulated dyes indicated the uptake of the complete nanoparticle and its cargo, with apparently minimal uptake of free dye molecules potentially released from the nanocarriers. In addition, these signals could be located both overlapping and not overlapping Lysotracker-dyed lysosome regions (Fig. 5a,c), suggesting the nanoparticles could be located both inside and outside of these organelles at the time point assayed. The same distribution could be observed for both plain and Tf-modified NPs, as well as NR- and Rh-loaded formulations.

## Discussion

Plain PLGA NPs produced by the methodology followed in this work are much widely characterized in the literature^11,17–19^. Specifically, on THC-PLGA NPs, a thorough characterization was previously reported by our team^11,12^, while the attachment of Tf to PLGA NPs through the carbodiimide strategy is also a commonly stablished approach^20–23^. Thus, the physicochemical characterization of the NPs produced in this work was focused on confirming the successful production of plain and Tf-modified formulations, to subsequently analyze the influence of the Tf modification on the later performance of the NPs.

Regarding previous works, the results of size and ZP of the plain PLGA NPs and THC-PLGA NPs here presented correlate with them^11,17–19^. The similar values of size and ZP of plain blank and plain THC-loaded PLGA formulations (PLGA NPs and THC-PLGA NPs) indicate a negligible drug adsorption of Δ^9^-THC onto the surface of NPs, being the majority of drug molecules incorporated into the NPs matrix^11^. In addition, the high EE values obtained are supported by the lipophilic character of the drug, which prevents its partitioning into the aqueous phase during the formulation process^11,24^.

Tf interaction with its receptor has been a widely established and exploited approach for an effective cellular specific uptake for several drugs aiming to target cancer cells and the blood brain barrier (BBB) mainly^25–28^, due to the differentiated high expression of the receptor in the target locations. In this work, Tf was coupled to plain PLGA NPs and THC-PLGA NPs via a carbodiimide strategy, a mild conjugation approach. High yields of Tf-coupling to the surface of NPs were obtained (Table 1), which are in agreement with the data reported in the scientific literature obtained with the same carbodiimide strategy^22^. The decrease in ZP values of NPs after Tf-coupling (Table 1) was expected since this chemical modification implies the participation of carboxylic acid groups on the surface of NPs that account for the negative surface charge prior to Tf-coupling, and thus this ZP modification further confirmed the attachment of Tf ligands to the NPs^22,29,30^.

The similarity between the EE values obtained with plain THC-PLGA NPs and Tf-THC-PLGA NPs indicate that no leakage of encapsulated drug is to be expected from the Tf-coupling procedure, most probably due to the same reduced probability of the partitioning of the lipophilic drug to the aqueous media responsible for the high EE and DL values^11,31,32^.

Concerns often raise in scientific discussion when it comes to label NPs by conjugating a fluorophore to their constitutive materials or by encapsulating it; a conjugated tracking molecule would provide information about where the carriers or their degradation products are located, but not about the delivered drug. On the other hand, encapsulated fluorophores would provide an approximation of the location and distribution expected from the encapsulated drug, but not about the fate of the carrier itself. In this work, a double labeling was proposed in order to differentiate between the tracking of the NP itself and its cargo; a fluorescent dye was conjugated to the backbone of the polymer to track the NPs, while additional dyes were encapsulated to illustrate the expected behavior of encapsulated drugs. Given the lipophilic character of Δ^9^-THC, a lipophilic dye NR was expected to provide a closer resemblance to the delivery process of these formulations. Nonetheless, both a lipophilic and a hydrophilic fluorophores were alternatively used to also allow for a more accurate comparison with the available literature, where all these labeling strategies are used indistinctively.

The same carbodiimide approach followed for Tf-conjugation was applied to the attachment of FITC to the polymer backbone prior to the production of NPs^33^, leading in this case to a fluorescently-labeled polymer. Although a higher mass ratio of coupled molecule with respect to the polymer was obtained with FITC (12.90 μg/mg of NPs for FITC vs. 5.1–7.8 μg/mg of NPs for Tf, Table 1), it should be noted that all the Tf molecules were conjugated to the surface of NPs, while FITC molecules where conjugated to the polymer before NPs production and thus a high percentage of molecules are expected to be finally located in the core of NPs. Nonetheless, considering the initial amount of FITC employed in the reaction, the final yield of product proved to be significantly less efficient than in the case of Tf (1.29% coupled FITC vs. 59–71% coupled Tf). However, the final mass ratio of conjugated FITC proved to be sufficient for NP tracking while not inducing differences in particle size after NPs production (Table 1). The ZP of FITC-NPs presented a decrease with respect to PLGA NPs (− 30 mV of PLGA NPs vs. − 18 mV of FITC-PLGA NPs, Table 1), which again is consistent with the implication of negatively charged PLGA carboxylic acid groups in the conjugation reaction. The ZP decrease due to the coupling of FITC to PLGA is consistent with the literature^34^, although comparisons should be made carefully when the conjugation is addressed with different coupling reactions.

For the double fluorescent labeling of NPs, NR was encapsulated in FITC-PLGA NPs as a lipophilic model molecule, and Rh as a hydrophilic model molecule^11,18,35^. The particle size and ZP values obtained with both NR- and Rh- loaded FITC-PLGA NPs were similar to blank FITC-PLGA NPs (p < 0.05) (Table 1), indicating that no molecules of fluorophores were expected to be located adsorbed on the surface of NPs but in the inner core of the carriers. The subsequent Tf-coupling to the surface of double-labeled NPs led to a decrease in ZP values with respect to FITC-PLGA NPs, either blank, NR- or Rh- loaded, which was once again expected and attributed to the neutralization of FITC-free carboxylic acid groups on particle surface after the attachment of the protein by conjugation. The lower values of CE% of Tf obtained with Tf-FITC-PLGA NPs with respect to Tf-PLGA NPs (Table 1) were attributed to the lower number of PLGA carboxylic acid groups available for Tf-conjugation, since they are also the substrate of the FITC-conjugating reaction that takes place in a step previous to NP production and Tf-coupling. In the specific case of Tf-Rh-FITC-PLGA NPs, which led to the lowest CE% value (51%), the decrease was attributed to residual molecules of Rh that could remain adsorbed on the surface of the NPs due to the partitioning to the aqueous phase during NPs production, effect that would be minimize in the case of NR due to its lipophilic character.

Finally, SEM imaging (Fig. 1) revealed a monodispersed population of spherical nanostructures in agreement with the DLS characterization results (Table 1) and our previous works on THC-PLGA NPs^11,12^, with no observable difference induced by the Tf coupling procedure or the presence of this moiety on the nanoparticle surface.

The biphasic release profile exhibited by both THC-PLGA-NPs and Tf-THC-PLGA (Fig. 2) was in agreement with previous results^11^, where the initial fast release phase was attributed to the loss of drug molecules located near the NPs surface and the following slower phase attributed to polymer degradation and/or drug diffusion through the polymeric matrix. The lack of significant differences between plain and Tf-modified NPs showed that the Tf-coupling procedure did not affect the release profile of the formulation, and hence probably neither the initial drug distribution in the polymer matrix. Again, this could be due to the lipophilic character of the drug and subsequently minimized probability of partitioning to the reaction aqueous medium^11,31^, which is also consistent with the preserved values of EE and DL.

Caco-2 cells are heterogeneous human epithelial colorectal adenocarcinoma cells employed as colorectal cancer (CRC) cell model expressing CB_1_ subtype cannabinoid receptors (CBRs)^36–39^ as well as cell model expressing Tf receptors^40–42^, and therefore they were selected as cell line for the studies. Cell viability assays confirmed the absence of toxicity of blank formulations, widely reported in the literature; PLGA is a biocompatible and biodegradable polymer and thus no toxicity was expected from PLGA-based nanoparticles^10,17,18,43^. On the other hand, free Δ^9^-THC, presented a statistically significant increase (p < 0.05) in cell viability at the first time point of incubation assayed (2 h) (Fig. 3) at 100 and 150 μM, which is consistent with previous results reported by our group^11,12^. It should be considered that, although cannabinoids are known to inhibit cell growth and induce apoptosis in tumour cells^44^, their effects on cell viability and proliferation are dependent on the specific cell culture assayed and the doses administrated^36,45,46^. Specifically, the dose employed is known to be crucial; low cannabinoid concentrations enhance cell proliferation in vitro through the stimulation of several pathways^4^. In in vivo conditions, this pro-tumorigenic effect is not observed, but the activation of these cellular pathways due to low cannabinoid concentration leads to the generation of resistance to THC antitumor action^4,47^. In this work, the encapsulation of Δ^9^-THC into PLGA NPs avoided this cell proliferation effect from free Δ^9^-THC in vitro at the first time point assayed (2 h, Fig. 3), while decreasing cell viability at the highest concentration tested (150 μM) at 24 h and 48 h compared to the free drug (24 h and 48 h, Fig. 3). These observations are in agreement with a previous work from our group^11^, where longer incubation times enabled a higher cytotoxic effect from plain THC-PLGA NPs compared to free Δ^9^-THC. These results are attributed to the controlled release of the drug from the nanoparticles, and hence lower but sustained delivered dose of cannabinoid to the cells^11^. Therefore, testing whether this effect is translated to in vivo would represent an interesting strategy against the development of resistance to Δ^9^-THC antitumor action.

Furthermore, Tf-THC-PLGA NPs provided a noticeable different result. This formulation did not only avoid the cell proliferation effect of free Δ^9^-THC at the first 2 h of incubation, but also provided decreased viability at 24 h (from 75 to 150 μ equivalent concentration), achieving the lowest cell viability values obtained in the study at 48 h (16% at the maximum dose analysed). These are considerably much shorter time periods than needed to achieve a similar effect with either Δ^9^-THC or plain THC-PLGA NPs (up to 3.5-fold)^11,12^.

For a better understanding of the mechanisms by which the Tf surface modification improved the cytotoxicity profile of the nanocarriers to such extent, assays with double-fluorescently-labeled NPs, either plain or Tf-modified, were carried out. When comparing the data reported in the literature, careful consideration must be paid to the details of experimental procedures; the variations on the methods of fluorescent labeling (encapsulation, chemical conjugation to the carrier constitutive materials, conjugation to encapsulated drugs, conjugation to targeting molecules, etc.), targeting molecule incorporation to the system (chemical coupling reaction, surface adsorption, dye encapsulation), the cell line assayed, incubation times and even the type of cell culture support (smooth or permeable, with or without additional chambers for drug diffusion), to mention a few, may induce differences in the output to a greater or lesser extent^48,49^. In this work, the double fluorescent labeling strategy presented, along with the use of either lipophilic or hydrophilic encapsulated dyes, and performing uptake studies with a wide time assay period, were meant to provide a wider perspective and comparison with scientific literature records.

Size, surface charge and hydrophobicity of NPs are widely acknowledged to influence the endocytic pathway in cells^50^. These parameters were considered similar for this matter between the formulations assayed in this piece of work (Table 1), and thus they were not regarded as influencing variables in the discussion of uptake studies.

Overall, the uptake of plain PLGA NPs has been identified in the literature as a combination of fluid phase pinocytosis and clathrin-mediated endocytosis. The first one would follow a linear progression while the latter is saturable, resulting in biphasic kinetics of cellular uptake^51^. The nanoparticles would be found later in lysosomes, from which they would escape to the cytosol as a result of the protonation of carboxylic groups in the acidic environment, exposing a hydrophobic surface that would interact with the membrane of the vesicles^52,53^. In addition, biphasic kinetics of PLGA NPs exocytosis have also been proposed, which would involve both endosomal escape to cytosol and exocytosis and also a recycling endosomal pathway, the latter avoiding passage through the cytosol^51^. On the other hand, Tf and Tf-coupled molecules interact with the Tf receptor and are subsequently internalized via clathrin-mediated endocytosis to follow the transferring recycling pathway, according to the literature^54,55^. Overall, two recycling pathways are differentiated; a faster one involving early endosomes and recycling endosomes, and a slower one involving lysosomes and the scape of the material to the cytosol followed by exocytosis^54^. As a general rule, recycled material follow both pathways with a preference related to its molecular weight and diffusion rate, where small molecules would follow the faster route and large molecules the slower one^51^. Hence, Tf-modified nanoparticles would be expected to follow the slow recycling pathway, also involving lysosomal scape to the cytosol as their non-modified counterparts, with the difference of the involvement of the Tf-receptor mediated interaction. Finally, it is worth considering that nanoparticles decorated with a receptor targeting ligand are generally expected to be uptaken at a higher rate^55^ than their non-modified counterparts.

The results of uptake studies from flow cytometry assays are depicted in Fig. 4. The uptake of FITC-PLGA NPs assumed from the fluorescent signal of FITC (Fig. 4a) revealed a time-dependent uptake of NPs with a rapid exponential phase in the first 2 h that then turned into a plateau, in agreement with the above discussed literature. The correspondent NR and Rh signals from the same formulations showed a similar profile (Fig. 4b), in accordance with published works. For instance, a linear time-dependent uptake profile was reported by Reix et al.^56^ with FITC-insulin loaded PLGA NPs in Caco-2 cultures in a 6 h study. Other sources reported different outcomes nonetheless, which could be ascribed to several experimental variables. Prior studies in our group presented a near 0% uptake values of NR-loaded plain PLGA NPs in Caco-2 cells in the first 6 h of incubation, that were increased at 24 h^11^. This could be probably due to differences in the methodology followed; in the present study, the cells were treated and fixed right at the end of the incubation period. However, in our previous studies^11^, the NPs suspension was replaced by culture media at the end of each incubation period, in order to treat and analyze the living cells at the end of the study. Exocytosis of nanoparticles from culture cells has been reported in several studies^51,57^, especially when the nanoparticles are removed from the incubation media and hence the concentration gradient is reversed^56,58^. Therefore, and regardless of the experimental specifics of each case, the maintenance of cells in NP-free media after incubation with the formulations could be overall considered a factor inducing decreased value of total uptake, due to nanoparticle exocytosis.

Surprisingly, Tf-FITC-PLGA NPs, on the other hand, presented a delayed internalization with respect to plain FITC-PLGA NPs (Fig. 4a), with intensity values around 10% after 2 h and maximum values at the final incubation period (48 h), therefore without a conclusive observation of uptake saturation within the time assayed. The corresponding signal from encapsulated dyes (Fig. 4b) showed a similar trend, differentiated from their non-Tf-modified counterparts, but with higher variations between values obtained with either NR or Rh. This observation could be due to some extent of dye release from the particles and its cell internalization dissociated from the carrier. In that case, higher internalization would be expected from NR compared to Rh, due to its lipophilic character, which would explain the slightly higher values of uptake from Tf-NR-FITC NPs vs. Tf-Rh-FITC NPs (Fig. 4b). Overall, in the case of Tf-modified formulations, the tracking of chemically linked dye (FITC, Fig. 4a) vs. encapsulated dyes (NR and Rh, Fig. 4b) led to a similar conclusion, i.e. delayed internalization of Tf-modified formulations vs. plain ones, albeit with differences in the obtained values in the case of loaded dyes. With regard to published literature, even though particle surface decoration with receptor ligands is generally reported to increase uptake rate^55^, some works agreed with decreased uptake due to Tf-surface modification^59^. Specifically, Zhang et al.^59^ reported coumarin-loaded, Tf-modified polyestyrene nanoparticles showing slower but more efficient internalization and faster transfer of the particles from the lysosome to the cytosol in HepG2 cells. In addition, Sahoo et al.^60^ reported a delayed but higher sustained uptake of coumarin and Taxol loaded, Tf-conjugated PLGA NPs vs. plain NPs in MCF-7 cells.

Overall, the clear difference in cell internalization rate of Tf-modified PLGA NPs vs. plain PLGA NPs is especially worth of attention, since it could play an important role in the improved cytotoxicity revealed by the MTT assays. It should be noted that CB_1_ receptors, whose activation is responsible for the antitumor effect of Δ^9^-THC^4,61^, are located at the surface of Caco-2 cells. Thus, the enhanced effect of Tf-THC-PLGA NPs could be based on a mechanism more complex than an increased intracellular accumulation of NPs, opposed to the underlying principle sustaining other strategies^62,63^. Initially, a higher residence time of the particles near the cell surface, and hence a sustained release of cannabinoid closer to its receptor, could be considered a reason for the higher efficacy of the formulation. On one hand, a slower internalization process via Tf receptor-mediated endocytosis vs. non-specific endocytosis could be responsible for this behavior. On the other hand, faster recycling and exocytosis of the particles through a Tf-related pathway could also be considered as potential cause for decreased particle cell accumulation, and hence increased presence in the external medium. Both hypotheses would need further studies to be conveniently assayed.

In an attempt to clarify the mechanisms involved in this interaction, further studies with endocytosis inhibitors were carried out. When hypertonic sucrose media was employed to inhibit clathrin-mediated endocytosis^64,65^, the uptake rate of Tf-FITC-PLGA NPs increased (Fig. 4c) at the first time points compared to standard conditions (Fig. 4a), while no significant differences were observed for plain FITC-PLGA NPs. Once again, tracking of NR and Rh signals (Fig. 4d) led to a similar general profile as with FITC tracking, albeit with differences in the obtained values at 1 h and 2 h. Given that the Tf internalization pathway through its Tf receptor involves the formation of clathrin-coated pits^50,62,63^, the results obtained supports that the delayed internalization observed for Tf-modified particles in standard conditions, potentially responsible for their enhanced antitumor effect, is due to a Tf-related cellular interaction. On the other hand, the increased uptake rate in the presence of sucrose points out that the nanoparticles are also internalized by alternative routes to clathrin-mediated endocytosis, which would allow for a faster cell uptake. Several endocytosis pathways have been suggested in the literature^52^, especially fluid phase pinocytosis^51^, as previously mentioned. While these pathways take place simultaneously in standard conditions, the density of the targeting ligand on nanoparticle surface would determine the predominant pathway^66^. It should be noted that, even when clathrin-mediated internalization is inhibited, including Tf-receptor mediated internalization, the uptake profile of Tf-modified nanoparticles still remained relatively slower than plain nanoparticles (Fig. 4c,d). Therefore, the presence of Tf moieties on particle surface may still exert an influence in non-clathrin-mediated internalization, which could be due to non-specific interactions related to its hydrophilic character, as opposed to plain FITC-PLGA NPs surface.

Following, the inhibition of caveolae-mediated endocytosis was performed by including genistein in the culture media. In this case, plain FITC-PLGA NPs presented a similar profile with respect to standard conditions (Fig. 4e vs. a), while the uptake rate of Tf-FITC-PLGA NPs was increased (Fig. 4e), resembling the results obtained with sucrose hypertonic media (Fig. 4c). Similarly, the values obtained through tracking of encapsulated dyes led to a similar profile to FITC tracking (Fig. 4f vs. e), with some differences between NR and Rh signals. Regarding plain nanoparticles, the results are supported by the previous work from Panyam et al.^67^ with HASMCs cells, where the the inhibition of actin polymerization by cytochalasin D did not affect the uptake of coumarin-6-loaded PLGA NPs, confirming no involvement of caveolae-mediated endocytosis. Regarding Tf-FITC-PLGA NPs, the observed increased uptake was surprising, since Tf-receptor internalization is known to be mediated by clathrin^54^, and even plain PLGA NPs are not expected to be internalized via caveolae-mediated mechanisms^51,53^. It should be noted that, although genistein does inhibit SV40 induced vesicle formation from caveolae, and thus it is widely used as inhibitor for caveolae-mediated endocytosis, this inhibition is not strictly specific for caveolae-mediated processes, since it has been reported to inhibit some clathrin-associated routes of receptors that imply tyrosine phosphorylation or F-actin recruitment^49^. Even though no studies are available in the literature to shed light on this specific matter for Tf-modified PLGA NPs interacting with Caco-2 cells, the closest comparison can be found, to the best of our knowledge, in the work of Chang et al.^68^. The authors assayed PLGA NPs coated with adsorbed FITC-Tf on their surface in a co-culture of brain endothelial cells and astrocytes as a blood brain barrier (BBB) cell model. The results showed an inhibition of Tf-adsorbed NPs uptake by filipin, an inhibitor of cholesterol-dependent mechanisms which involve caveolae-mediated uptake as well as other clathrin-mediated mechanisms such as micropinocytosis^49^.

Finally, the near 0% values of fluorescence obtained with the inhibition of energy-dependent processes (Fig. 4g,h) confirmed that the internalization of all the formulations assayed took place by energy-dependent mechanisms irrespective of the Tf-functionalization^69,70^.

Qualitative assessment of the interaction of NPs with the cells by fluorescent microscopy showed a similar distribution of both FITC and NR or Rh signals in the cell culture (Fig. 5a), suggesting that the encapsulated dyes were internalized in the cells still loaded in the nanoparticles, with minimal absorption of released dye. It should be considered nonetheless that microscopy imaging provides only qualitative information, and hence some degree of free dye absorption, as flow cytometry results suggested, should not be discarded. Still, in the light of these results, tracking of encapsulated dyes was overall confirmed to be a valid strategy to track the nanoparticle structure inside the cells in this case. In addition, the higher intensity of fluorescence obtained in cells incubated with plain formulations vs. Tf-modified formulations (Fig. 5a,b) was in agreement with the delayed internalization observed by flow cytometry in the case of Tf-modified NPs. On the other hand, the nanoparticles were located both overlapping and not-overlapping with Lysotracker-dyed regions both with plain and Tf-modified formulations (Fig. 5a,c), indicating partial distribution of the nanocarriers inside and outside lysosomes, in agreement with previous studies on PLGA NPs^51^. It should be noted that Lysotracker dyed acidic organelles, and thus it could not be assayed whether nanoparticles outside of lysosomes were located inside early or recycling endosomes, or in the cytosol. However, even though fluorescent microscopy imaging did not clarify further the preferred internalization and trafficking pathway of the nanoparticles, it did confirm the involvement of lysosome-associated routes, which is in agreement with the results obtained from flow cytometry analysis.

To sum up, in light of the flow cytometry analysis results and fluorescent microscopy imaging, the internalization mechanism potentially involved in the improved cytotoxic effect of Δ^9^-THC-loaded Tf-PLGA NPs seemed dependent on cholesterol-associated and clathrin-mediated mechanisms, which apply for endocytosis of receptors that are dependent on tyrosine phosphorylation, including the Tf receptor^54^. In addition, both fluorescent labeling strategies, either chemical linkage or dye encapsulation, allowed to withdraw overall similar conclusions, although the latter was subject to differences in the values obtained in each case. Hence, chemical linkage of the fluorophore seems the best suited approach to track the nanoparticle itself, while dye encapsulation seems to provide a better approximation to the fate of the loaded drug.

It should be noted that the presented cell studies were carried out in FBS-free medium, in order to minimize variability from growing cells. The presence of proteins is known to strongly influence the interaction of nanocarriers with cells due to the formation of the protein corona, which is considered nowadays a main factor for discordance between in vitro and in vivo results^71,72^. However, in vitro-formed corona has been reported to be radically different from in vivo conditions, thus being the use of FBS strongly discouraged in these studies^72^. Specifically, Tf-modified polyestyrene nanoparticles were recently reported to lose their targeting ability with the formation of an in vitro corona, but retain it in vivo with high affinity to the target cells^59^. Hence, a similar behavior would be plausible in the case of Δ^9^-THC-loaded Tf-PLGA NPs. Nonetheless, additional studies should be performed to further asses the interaction mechanism of the proposed formulation with the target cells, as well as testing it in vivo.

## Conclusions

Δ^9^-THC has been reported to inhibit tumor angiogenesis and cell growth in malignant tissues. However, its unfavorable physicochemical and biopharmaceutical properties, along with resistance mechanisms related to dosing, require the development of an adequate drug delivery system. In this work, plain and Tf-modified Δ^9^-THC PLGA NPs, and FITC-conjugated, NR- and Rh-loaded PLGA NPs were successfully produced and characterized. All the formulations resulted in monodispersed nanoparticles of size around 300 nm and negative zeta potential. THC-loaded formulations showed an EE over 90%, 4.6% DL and a biphasic extended release. Overall, plain and Tf-modified PLGA NPs proved to be a valuable carrier for loading THC with reproducibility. Following, Tf-THC PLGA NPs decreased cell viability down to 17% vs. 88% with plain THC-PLGA NPs, confirming the improvement of the formulation with the introduction of the Tf moiety. In addition, flow cytometry analysis revealed that Tf-modified NPs were internalized at a significantly slower rate than plain THC PLGA NPs, suggesting that a potential maximization of the amount of drug released in a sustained manner at the surface of cells bearing cannabinoid receptors could be the reason for the improved efficacy of the formulation. Moreover, uptake studies in the presence of inhibitors and fluorescent microscopy studies suggested that both types of NPs were internalized through cholesterol-associated and clathrin-mediated mechanisms. Additionally, florescent microscopy imaging revealed a higher uptake of plain NPs vs. Tf-modified formulations at the assayed points, supporting the results obtained by flow cytometry. Furthermore, comparison of fluorescent signals from dyes either chemically linked to the nanoparticle polymer or loaded into the nanoparticles simultaneously provided complementary information on the internalization process.

Overall, Tf-modification of PLGA NPs seemed a highly promising approach for Δ^9^-THC-based antitumor therapies, aiming at a prolonged action of the carrier at the target cell surface. Moreover, the translation of this strategy to the delivery of alternative active pharmaceutical ingredients with pharmacological targets on the surface of cells could lead to advances in related therapies. Further characterization studies on this interaction, including the monitoring of receptor expression on the cells throughout NPs treatment, would provide valuable insights. In addition, dual-labelling of NPs through different strategies, either chemical conjugation or dye loading, and including both hydrophilic and lipophilic dyes, resulted in an interesting approach that could be further exploited to investigate the conjunct behaviour of the nanocarriers and the associated drug.

## Methods

### Materials

Δ^9^-THC (99.3%) was provided by THC Pharma GmbH, (Frankfurt/Main, Germany). Poly(DL-lactide-co-glycolide) (PLGA 50:50) Resomer®RG 502H (Mw: 12,000; inherent viscosity: 0.19 dl/g) was obtained from Evonik Industries AG (Essen, Germany). Poly(vinyl alchohol) (87–90% hydrolyzed, Mw: 30,000–70,000) PVA (5.6 cps), Nile Red (74.95% Carbon–8.65% Nitrogen (Elemental analysis), Rhodamine B (99%), human transferrin (Tf) (≥ 98%), *N*-(3-Dimethylaminopropyl)-*N*′-ethylcarbodiimide hydrochloride (EDC∙HCl) (≥ 98%), *N*-Hydroxysuccinimide (NHS) (≥ 99.4%), fluorescein isothiocyanate isomer I (FITC) (≥ 90%), sucrose and genistein were purchased from Sigma-Aldrich (St Louis, MO; USA). Glycerol (98.0–101.0%) was obtained from Acofarma Distribución S.A. (Barcelona, Spain). Trehalose (≥ 98%) was obtained from VWR International Eurolab S.L. (Barcelona, Spain). Solvents used ethyl acetate (EA), acetonitrile, methanol, pyridine, dimetylsulfoxide (DMSO) (HPLC-grade) and acetic acid were purchased from Panreac Química (Barcelona, Spain). Deionized and filtered water was used in all the experiments (Milli-Q Academic, Millipore, Molsheim, France).

The RP-HPLC analysis was carried out with a Hitachi LaChrom^®^ (D-7000) Series HPLC system equipped with a L-7200 automatic injector, a D-7000 interphase, a L-7100 quaternary pump and a DAD UV–vis L-7455 detector. A Waters column (3 µm, 4.6 × 100 mm, Milford, MA, USA) maintained at 25.0 ± 0.1 °C (L-2350 column oven, Elite LaChrom^®^) was used in this analysis. Data collection and calculation were carried out using HSM D-7000 LaChrom^®^ software by Merck-Hitachi (Darmstadt, Germany).

For cell line experiments, human colon adenocarcinoma cells (Caco-2) cells were obtained from the European Collection of Cell Cultures (ECACC); number 86010202 (Salisbury, UK). Minimum Essential Medium Eagle (MEM with Earle´s salts without L-glutamine), sodium pyruvate (100 mM), MEM non-essential amino acids (100×), L-glutamine (200 mM (100×)) and fetal bovine serum (FBS) (non-USA origin, sterile-filtered, suitable for cell culture) were obtained from Biowest (Biowest, Nuaillé, France). Gentamicin (10 mg/mL) was purchased from Gibco^®^ Life Technologies Corporation (NY, USA). Trypsin/EDTA (1×, sterile-filtered, BioReagent, 500 BAEE units porcine trypsin and 180 µg EDTA · 4Na), MTT (3-(4,5-Dimethylthiazol-2-yl)-2,5-diphenyltetrazolium bromide, a yellow tetrazole) (BioReagent, ≥ 97.5%); sodium dodecyl sulphate (SDS) (≥ 99.0%) and paraformaldehyde (PFA) (95%) were purchased from Sigma-Aldrich (St Louis, MO, USA). LysoTracker^®^ Blue DND-22 was obtained from Life Technologies, (Thermo Scientific Inc., Spain). Phosphate buffered saline (PBS) pH 7.4, was obtained from Biochemica AppliChem (Darmstadt, Germany).

### Production of PLGA NPs

PLGA NPs were produced by a modified emulsion solvent evaporation method (SEV)^73^. For that purpose, 2 mL of a 4% w/v PLGA solution in EA was added to 15 mL of an aqueous 0.5% w/v PVA solution, and the mixture was immediately homogenized at 25.000 rpm (Heidolph homogenizer DIAX 900, Germnay*)* for 1 min. Following, the resultant emulsion was kept under stirring for 4 h until complete evaporation of the solvent. The resultant nanoparticles were washed by adding 15 mL of water to the formulation and centrifuging for 30 min at 10,000 rpm, 4 °C (Eppendorf 504R centrifuge, Eppendorf AG, Germany). The supernatant was kept for further studies while the particles pellet was resuspended in 1 mL of a 5% w/v trehalose solution as cryoprotectant with the help of a vortex and freeze-dried (− 80 ± 0.5 °C and 0.057 mbar; Cryodos freeze-dryer, Telstar Industrial S.L., Spain) to obtain a fine powder. In the case of Δ^9^-THC-loaded nanoparticles (THC-PLGA NPs), Δ^9^-THC was co-dissolved in the PLGA solution at a concentration of 5% w/w referred to the amount of polymer, as previously reported^12^.

### Preparation of FITC-labeled PLGA

FITC was covalently coupled to PLGA by the carbodiimide method reported elsewhere^33^ with some modification. For the activation of the carboxylic chemical groups of the polymer, 5 mL of each, 1.5% w/v NHS, 2.0% w/v EDC and 20% w/v PLGA solutions in DCM were mixed and incubated for 2 h at room temperature (RT) under mild agitation. Then, 40 mg of FITC dissolved in 500 μL of pyridine and 500 μL of DCM were added to the previous solution, and the mixture was incubated for 2 h in the dark at RT and under agitation, followed by overnight incubation in the dark at 4 °C. The modified PLGA was purified and recovered by inducing its precipitation, adding the solution in a dropwise manner to methanol kept under stirring at 4 °C. Several washes in methanol were applied to the polymer until the supernatant was clear of detectable fluorescence. Finally, the polymer was washed in water for the removal of methanol, freeze-dried (− 80 ± 0.5 °C and 0.057 mbar; Cryodos freeze-dryer, Telstar Industrial S.L., Spain) and stored in the dark at 4 °C.

### Coupling of Tf to the surface of NPs

Tf was conjugated on the surface of PLGA NPs and THC-PLGA NPs (Tf-PLGA NPs and Tf-THC-PLGA NPs, respectively) by the two-step carbodiimide reaction reported elsewhere^22^ with some modification, involving the obtention of an amide bond between the carboxylic end group of PLGA on the surface of NPs and the amine terminal group from Tf. A scheme illustrating the reaction can be found in Supplementary Fig. 1. Briefly, 10 mg of lyophilized NPs were dispersed in 5 mL of PBS along with 250 μL of a 1 mg/mL solution of EDC in PBS and 250 μL of a 1 mg/mL solution of NHS in PBS, in order to activate the carboxylic acid groups of the surface of NPs. The mixture was stirred for 4 h at RT and then the particles were collected by centrifugation (30 min at 10,000 rpm, 4 °C, Eppendorf 504R centrifuge, Eppendorf AG, Germany). Thereafter, the activated particles were dispersed in 2 mL of PBS, and 100 μL of a solution of Tf in PBS at 1 mg/mL were added to the suspension in a dropwise manner. The mixture was further incubated for 2 h at RT under stirring followed by overnight incubation at 4 °C. The suspension was finally collected by centrifugation (30 min at 10,000 rpm, 4 °C, Eppendorf 504R centrifuge, Eppendorf AG, Germany) to remove unreacted molecules, suspended in 5% w/v trehalose and freeze. The supernatant was analyzed for Tf conjugation efficacy.

### Production of doubled-labeled fluorescent PLGA NPs

FITC-labeled PLGA NPs (FITC-PLGA NPs) were produced by the SEV method as described in the prior section, employing FITC-PLGA instead of plain PLGA. To prepare Nile Red-labeled nanoparticles (NR-FITC PLGA NPs), 100 μL of a Nile Red solution 1 mM in DMSO were added to the FITC-PLGA solution. Finally, in order to label the particles with Rhodamine B (Rh-FITC PLGA NPs), a double-emulsion solvent evaporation method (DE/SEV) was employed^73^: briefly, the 2 mL FITC-PLGA solution in EA was placed in an ultrasonic bath (intensity, name, brand, country) and 100 μL of a 1 mg/mL solution of Rhodamine B in 0.5% w/v PVA solution was added dropwise under sonication. The mixture was covered and maintained in the ultrasonic bath for 1 min (50 kHz, sonic power: 50 W; Selecta S.A., Barcelona, Spain) in order to produce a water-in-oil (w/o) emulsion. This emulsion was subsequently added to 15 mL of a 0.5% w/v PVA aqueous solution and manipulated as described above for the SEV procedure. To produce Tf-conjugated Nile Red- or Rhodamine B-loaded FITC-PLGA NPs (Tf-NR-FITC NPs and Tf-Rh-FITC PLGA NPs, respectively), NR-FITC-PLGA NPs and Rh-FITC-PLGA NPs were used instead of plain PLGA-NPs to perform the Tf-coupling reaction above described.

### Characterization methods

The mean diameter and size distribution of NPs were measured by laser light scattering, and their zeta potential (ZP) by Laser Doppler, both in a Zetasizer Nano ZS90 (Malvern Instruments Ltd., Malvern UK). Measurements were carried out in triplicate by diluting an aliquot of recently prepared NPs suspensions with purified water at 25 ± 0.5 °C.

The morphological characterization of the NPs was performed by image analysis obtained by scanning electron microscopy (SEM) in a FEI TENEO microscope. Particles were cover with a 8–9 nm Pd/Pt shell under vacuum (Leica EM SCD500).

### Δ^9^-THC encapsulation efficiency and loading capacity

The determination of the drug content of the NPs was carried out as previously described^12^ . Around 5 mg of lyophilized NPs were accurately weighed using a high-precision analytical balance (*d* = 0.01 mg; Model CP 225D; Sartorius AG, Göttingen, Germany). Following, 5 mL of acetone were added and the mixture was accurately vortexed to dissolve the particles in the organic phase. Acetone was then evaporated using a rotavapor (Büchi R/210, BüchiLabortechnik AG, Flawil, Switzerland). Next, 1 mL of ethanol was added and the Δ^9^-THC content solved under sonication (Branson 50 W, Branson Ultrasonics Corporation, Danbury, CT, USA) and vortexing for 5 min. The obtained drug solution, was then filtered (0.22 µm syringe filter, Millex® GV, Millipore, Barcelona, Spain) and injected into the HPLC system for Δ^9^-THC detection following a validated method^10^.

The drug content was expressed as *entrapment efficiency* (*EE %*) and *drug loading (DL %)* following the Eqs. (1 and 2):

1 $$EE\%=\left(\frac{actual\,amount\,of\,\Delta 9-THC\,loaded\,in\,NPs }{theoretical\,amount\,of\,\Delta 9-THC\,in\,NPs}\right)\times 100$$

2 $$DL\%=\left(\frac{mass\,of\,\Delta 9-THC\,in\,NPs }{mass\,of\,NPs\,recovered}\right)\times 100$$

### In vitro drug release

4 mg of lyophilized NPs were suspended in 15 mL of phosphate buffered saline, PBS, pH = 7.4 ± 0.1 containing 0.1% (w/v) of Tween^®^ 80 to assure the sink conditions^18^. PBS was maintained at 37.0 ± 0.5 °C and under mechanical stirring (100 rpm, Unitronic OR horizontal shaker, Selecta S.A., Spain) during the release experiments. Aliquots (500 µL) were withdrawn at fixed time intervals, centrifuged at 10,000 rpm during 5 min (Eppendorf 504R centrifuge, Eppendorf AG, Germany), and the supernatants were filtered with a 0.22 µm syringe filter (Millex®-GV, Millipore, Spain). Finally, 20 µL of each sample were injected into the HPLC system to evaluate the amount of Δ^9^-THC released into the PBS medium. The amount of Δ^9^-THC released at each time was determined by HPLC as previously described^11^. After sampling, an equal volume of release medium, maintained at the same temperature, was added to maintain sink conditions.

### Conjugation efficacy of Tf and FITC

The efficacy of the conjugation of Tf to NPs was studied by an indirect calculation, from the analysis of the supernatant obtained in the last step of the conjugation process with a Micro BCA Protein Assay Kit (Thermo Scientific Inc., Spain)^69^. The conjugation of FITC to NPs was also analyzed by and indirect method by measuring the fluorescence remaining in methanol supernatants from the purification of FITC-PLGA at excitation and emission wavelengths of 495 nm and 520 nm respectively. Both studies were carried out in a microplate reader (Synergy™ HT multimode microplate reader, BioTek Instruments Inc., USA).

The conjugation efficacy was expressed as CE% following Eq. (3):

3 $$CE\%=\left(\frac{Initial\,amount\,of\,ligand-Amount\,of\,ligand\,in\,the\,supernatant }{Initial\,amount\,of\,ligand}\right)\times 100$$

In addition, the ratio of mass of coupled molecule per unit of mass of NPs was calculated as well.

### Cell studies

#### Cell culture

Caco-2 cells were cultivated as previously reported^11,17,18^. MEM media (with Earle’s salts without L-glutamine) was employed as culture medium, supplemented with 10% v/v FBS, 1% v/v sodium pyruvate, MEM non-essential aminoacids and L-glutamine, and 0.1% v/v gentamicin. Cell cultures were then kept at 37.0 ± 0.5 °C in a humidified atmosphere of 5% CO_2_ in air (water-jacketed US autoflow NU-4750 automatic CO_2_ incubator, NuAire Inc., USA). The medium was replenished every other day and cells were subcultured after reached confluence.

#### Cellular toxicity

The cytotoxicity of free Δ^9^-THC, plain blank PLGA NPs, blank Tf-PLGA NPs, Δ^9^-THC-PLGA NPs and Tf-THC-PLGA NPs was tested by triplicate by the MTT proliferation assay as previously reported^11^. For this purpose, Caco-2 cells under exponential growth phase were seeded into 96-well plates at a density of 7 × 10^5^ (Nunclon®, Thermo Fisher Scientific Inc., USA) with free-FBS media, and incubated for 24 h to allow for cell attachment. Then, the nanoparticles were added to the cell culture, reaching a final NPs concentration in the range of 0.16 -0.96 mg/mL in the culturing media, corresponding to a Δ^9^-THC equivalent dose of 25–150 μM. Free Δ^9^-THC was added at the same concentration, previously dissolved in acetone and ensuring a final concentration of acetone in the culture media below 0.1% v/v. Untreated Caco-2 cells were used as negative control, and cells treated with DMSO (toxic at concentration over 25 μM) were taken as positive control. After a period of incubation of 2, 24 and 48 h, the medium was removed, the wells were washed twice with sterile PBS and then 125 μL of an MTT solution (1 mg/mL of final MTT concentration in culture media) were added to the wells. The plates were further incubated for 2 h, when 80 μL of a 20% w/v solution of SDS in 0.2 M HCl were added to dissolve the MTT crystals, upon incubation during 24 h at 37.0 ± 0.5 °C in the dark (water-jacketed US autoflow NU-4750 automatic CO_2_ incubator, NuAire Inc., USA). Finally, cell viability was quantified by measuring the optical density (OD) of each well at 540 nm (Synergy™ HT multimode microplate reader, BioTek Instruments Inc., USA).

#### Flow cytometry analysis of NPs uptake: energy dependence and inhibition

Quantitative cellular uptake of fluorescently labeled formulations was carried out following previously reported procedures^11,18^ with additional modifications. Caco-2 cells were cultured in T25 flasks (Nunclon®, Thermo Fisher Scientific Inc., USA) with FBS-supplemented media as described in the “Cell culture” section, until reaching cell confluence (1 × 10^6^ cells/flask aprox.). Following, the media was withdrawn and cells were pre-incubated for 30 min with either sucrose at 200 mM for the inhibition of clathrin-dependent uptake mechanisms or genistein at 200 μM for the inhibition of caveolae-dependent mechanisms^74–77^. In addition, energy-dependent mechanisms were inhibited by pre-incubating the cells at 4 °C for 30 min^69,70^. Following, the media was replaced by a suspension of 0.5 mg/mL of NR-FITC PLGA NPs, Rh-FITC-PLGA NPs, Tf-NR-FITC PLGA NPs or Tf-Rh-FITC-PLGA NPs. The 0.5 mg/mL concentration of NPs, corresponding to 75 μM of Δ9-THC, was selected on the basis of: (i) having been assayed by MTT, proving the absence of toxicity from blank NPs with a safety margin (Fig. 3); (ii) enabling cytotoxicity effect when loading Δ9-THC and bearing Tf on NPs surface vs. plain NPs (Fig. 3); and (iii) allowing to detect and track the signal from all the fluorophores at the ratios employed in the production of the NPs. The NPs were added to the cells suspended in FBS-free culture media, either alone (control) or in the presence of 200 mM sucrose, 200 μM genistein, or alone at 4 °C (4^4^ combinations). After incubation periods of 0.5; 1; 2; 5; 24 and 48 h, the media was withdrawn and the cell layers washed twice with PBS to eliminate unattached NPs, and trypsinized for cell detachment. Thereafter, culture media was added to the cell suspension to neutralize trypsin, and the cells were transferred to a 15 mL tube to be centrifuged (5 min; 300 g; 25 °C). Then, the supernatant was carefully removed and the cell pellet suspended in 1 mL culture media with smooth vortexing. Next, 3 mL of PFA 4% w/v in PBS were dropwise added to the cell suspension while vortexing smoothly (final PFA concentration 3% w/v) to fix the cells^69^. The cell suspensions were left at RT for 15 min and then stored at 4 °C in the dark until flow cytometry analysis. Finally, and prior to flow cytometry analysis, the cell suspensions were centrifuged (5 min; 300 g; 25 °C), the supernatant removed and the cell pellet suspended in 1 mL of PBS to attain a concentration of cells of nearly 1 × 10^6^ cells/mL for optimum analysis, and the amount of fluorescence was then measured (Cytomics FC 500 MPL flow cytometer, Beckman Coulter Inc., USA)^11,18^. Negative control of reference for 0% fluorescence intensity was set by analyzing cells without NP treatment with a minimum of 10,000 cells counted per sample. A collection gate was subjectively chosen from the distribution in the side scatter (SS) vs. forward scatter (FS) dot plot to exclude cellular fragments and debris, and the obtained level of fluorescence was set as 0% in channels FL1 (corresponding to FITC signal) and FL2 (corresponding to Nile Red and Rhodamine B signal), yielding the respective regions for quantification of the normalized fluorescence intensity (C and E respectively) (Supplementary Fig. S2)^78^.

#### Intracellular tracking of NPs and their cargo

Caco-2 cells were cultured over previously sterilized circular coverslips placed on 12-well plates (Nunclon^®^, Thermo Fisher Scientific Inc., USA), at a density of 5 × 10^4^ cells/well with FBS-free media during 24 h to allow for cell attachment. Then, the media was replaced by a 0.5 mg/mL suspension of either NR-FITC PLGA NPs, Rh-FITC-PLGA NPs, Tf-NR-FITC PLGA NPs or Tf-Rh-FITC-PLGA NPs in FBS-free culture media. After 2 h of incubation, the media was withdrawn and the cells washed twice with sterile PBS. A solution of Lysotracker Blue 75 nM in FBS-free media was added to the wells and incubated for 30 min, followed by withdrawal of the medium and washing of the cell monolayer with sterile PBS (× 2). The coverslips were finally mounted by placing them cell-side down on a drop of PBS-glycerol 20:80 on glass slides and immediately visualized by fluorescent microscopy (Leica DM2000 coupled to color CCD camera Leica DFC425C).

### Statistical analysis

Statistical analysis (Prism 8, GraphPad Software, LLC) was performed using Student’s *t*-test and one-way analysis of variance (ANOVA) to define the significance between two groups and between more than two groups, respectively. All experiments were performed by triplicate. Results are presented as mean value ± standard deviation (SD). Data with p < 0.05 was considered significant.

## Supplementary Information

Supplementary Information.

## References

[1] Velasco G, Sánchez C, Guzmán M (2012). Towards the use of cannabinoids as antitumour agents. Nat. Rev. Cancer.

[2] Aviello G (2012). Chemopreventive effect of the non-psychotropic phytocannabinoid cannabidiol on experimental colon cancer. J. Mol. Med..

[3] Rocha FCM, Dos Santos Júnior JG, Stefano SC, Da Silveira DX (2014). Systematic review of the literature on clinical and experimental trials on the antitumor effects of cannabinoids in gliomas. J. Neuro-Oncol..

[4] Velasco G, Sánchez C, Guzmán M (2016). Anticancer mechanisms of cannabinoids. Curr. Oncol..

[5] Brownjohn PW, Ashto JC (2012). Cannabinoids and neuropathic pain. Neuropathic Pain.

[6] Szczesniak AM, Kelly MEM, Whynot S, Shek PN, Hung O (2006). Ocular hypotensive effects of an intratracheally delivered liposomal Δ9-tetrahydrocannabinol preparation in rats. J. Ocul. Pharmacol. Ther..

[7] Murgia S (2013). Characterization of the Solutol® HS15/water phase diagram and the impact of the Δ9-tetrahydrocannabinol solubilization. J. Colloid Interface Sci..

[8] Cherniakov I (2017). Piperine-pro-nanolipospheres as a novel oral delivery system of cannabinoids: Pharmacokinetic evaluation in healthy volunteers in comparison to buccal spray administration. J. Control. Release.

[9] Izgelov D, Shmoeli E, Domb AJ, Hoffman A (2020). The effect of medium chain and long chain triglycerides incorporated in self-nano emulsifying drug delivery systems on oral absorption of cannabinoids in rats. Int. J. Pharm..

[10] Martín-Banderas L (2012). Cannabinoid derivate-loaded PLGA nanocarriers for oral administration: Formulation, characterization and cytotoxicity studies. Int. J. Nanomed..

[11] Martín-Banderas L (2014). Engineering of δ9-tetrahydrocannabinol delivery systems based on surface modified-PLGA nanoplatforms. Colloids Surf. B Biointerf..

[12] Martín-Banderas L (2015). In vitro and in vivo evaluation of Δ9-tetrahidrocannabinol/PLGA nanoparticles for cancer chemotherapy. Int. J. Pharm..

[13] Yoshikawa T, Pardridge WM (1992). Biotin delivery to brain with a covalent conjugate of avidin and a monoclonal antibody to the transferrin receptor. J. Pharmacol. Exp. Ther..

[14] Yan F (2013). Transferrin-conjugated, fluorescein-loaded magnetic nanoparticles for targeted delivery across the blood-brain barrier. J. Mater. Sci. Mater. Med..

[15] Ulbrich K, Hekmatara T, Herbert E, Kreuter J (2009). Transferrin- and transferrin-receptor-antibody-modified nanoparticles enable drug delivery across the blood-brain barrier (BBB). Eur. J. Pharm. Biopharm..

[16] Sahoo SKSK, Ma W, Labhasetwar V (2004). Efficacy of transferrin-conjugated paclitaxel-loaded nanoparticles in a murine model of prostate cancer. Int. J. Cancer.

[17] Durán-Lobato M, Martín-Banderas L, Gonçalves LMD, Fernández-Arévalo M, Almeida AJ (2015). Comparative study of chitosan- and PEG-coated lipid and PLGA nanoparticles as oral delivery systems for cannabinoids. J. Nanoparticle Res..

[18] Durán-Lobato M (2014). Enhanced cellular uptake and biodistribution of a synthetic cannabinoid loaded in surface-modified poly(lactic-co-glycolic acid) nanoparticles. J. Biomed. Nanotechnol..

[19] Martín-Banderas L (2013). Biocompatible gemcitabine-based nanomedicine engineered by Flow Focusing® for efficient antitumor activity. Int. J. Pharm..

[20] Kuo YC, Lin PI, Wang CC (2011). Targeting nevirapine delivery across human brain microvascular endothelial cells using transferrin-grafted poly(lactide-co-glycolide) nanoparticles. Nanomedicine.

[21] Das M, Dilnawaz F, Sahoo SK (2011). Targeted nutlin-3a loaded nanoparticles inhibiting p53-MDM2 interaction: Novel strategy for breast cancer therapy. Nanomedicine.

[22] Cui Y, Xu Q, Chow PKH, Wang D, Wang CH (2013). Transferrin-conjugated magnetic silica PLGA nanoparticles loaded with doxorubicin and paclitaxel for brain glioma treatment. Biomaterials.

[23] Cayero-Otero MD (2019). In vivo biodistribution of venlafaxine-PLGA nanoparticles for brain delivery: plain vs. functionalized nanoparticles. Expert Opin. Drug Deliv..

[24] Barichello JM, Morishita M, Takayama K, Nagai T (1999). Encapsulation of hydrophilic and lipophilic drugs in PLGA nanoparticles by the nanoprecipitation method. Drug Dev. Ind. Pharm..

[25] Biffi S, Voltan R, Bortot B, Zauli G, Secchiero P (2019). Actively targeted nanocarriers for drug delivery to cancer cells. Expert Opin. Drug Deliv..

[26] Marcos-Contreras OA (2020). Selective targeting of nanomedicine to inflamed cerebral vasculature to enhance the blood–brain barrier. Proc. Natl. Acad. Sci. U.S.A..

[27] Alzhrani R (2020). Improving the therapeutic efficiency of noncoding RNAs in cancers using targeted drug delivery systems. Drug Discov. Today.

[28] Kurmi BD, Patel P, Paliwal R, Paliwal SR (2020). Molecular approaches for targeted drug delivery towards cancer: A concise review with respect to nanotechnology. J. Drug Deliv. Sci. Technol..

[29] Sahoo SK, Labhasetwar V (2005). Enhanced antiproliferative activity of transferrin-conjugated paclitaxel-loaded nanoparticles is mediated via sustained intracellular drug retention. Mol. Pharm..

[30] Shah N, Chaudhari K, Dantuluri P, Murthy RSRSR, Das S (2009). Paclitaxel-loaded PLGA nanoparticles surface modified with transferrin and Pluronic®P85, an in vitro cell line and in vivo biodistribution studies on rat model. J. Drug Target..

[31] Chronopoulou L (2013). Chitosan-coated PLGA nanoparticles: A sustained drug release strategy for cell cultures. Colloids Surf. B Biointerfaces.

[32] Chen J, Li S, Shen Q (2012). Folic acid and cell-penetrating peptide conjugated PLGA-PEG bifunctional nanoparticles for vincristine sulfate delivery. Eur. J. Pharm. Sci..

[33] Yin YS, Chen DW, Qiao MX, Wei XY, Hu HY (2007). Lectin-conjugated PLGA nanoparticles loaded with thymopentin: Ex vivo bioadhesion and in vivo biodistribution. J. Control. Release.

[34] Yang X (2016). Uptake and bioconversion of stereoisomeric dipeptide prodrugs of ganciclovir by nanoparticulate carriers in corneal epithelial cells. Drug Deliv..

[35] Trapani A (2010). A comparative study of chitosan and chitosan/cyclodextrin nanoparticles as potential carriers for the oral delivery of small peptides. Eur. J. Pharm. Biopharm..

[36] Ligresti A (2003). Possible endocannabinoid control of colorectal cancer growth. Gastroenterology.

[37] Alhamoruni A, Lee ACC, Wright KLL, Larvin M, O’Sullivan SEE (2010). Pharmacological effects of cannabinoids on the Caco-2 cell culture model of intestinal permeability. J. Pharmacol. Exp. Ther..

[38] Alhamoruni A, Wright KL, Larvin M, O’Sullivan SE (2012). Cannabinoids mediate opposing effects on inflammation-induced intestinal permeability. Br. J. Pharmacol..

[39] Karwad MA (2019). Endocannabinoids and endocannabinoid-like compounds modulate hypoxia-induced permeability in CaCo-2 cells via CB1, TRPV1, and PPARα. Biochem. Pharmacol..

[40] Saini P, Ganugula R, Arora M, Kumar MNVR (2016). The next generation non-competitive active polyester nanosystems for transferrin receptor-mediated peroral transport utilizing gambogic acid as a ligand. Sci. Rep..

[41] Ganugula R, Arora M, Guada M, Saini P, Kumar MNVR (2017). Noncompetitive active transport exploiting intestinal transferrin receptors for oral delivery of proteins by tunable nanoplatform. ACS Macro Lett..

[42] Yong JM, Mantaj J, Cheng Y, Vllasaliu D (2019). Delivery of nanoparticles across the intestinal epithelium via the transferrin transport pathway. Pharmaceutics.

[43] Martin-Banderas L (2013). Functional PLGA NPs for oral drug delivery: Recent strategies and developments. Mini Rev. Med. Chem..

[44] Gustafsson SB, Lindgren T, Jonsson M, Jacobsson SOP (2009). Cannabinoid receptor-independent cytotoxic effects of cannabinoids in human colorectal carcinoma cells: Synergism with 5-fluorouracil. Cancer Chemother. Pharmacol..

[45] Lopez-Rodriguez M, Viso A, Ortega-Gutierrez S, Diaz-Laviadac I (2012). Involvement of cannabinoids in cellular proliferation. Mini-Reviews Med. Chem..

[46] Hart S, Fischer OM, Ullrich A (2004). Cannabinoids induce cancer cell proliferation via tumor necrosis factor α-converting enzyme (TACE/ADAM17)-mediated transactivation of the epidermal growth factor receptor. Cancer Res..

[47] López-Valero I (2018). Optimization of a preclinical therapy of cannabinoids in combination with temozolomide against glioma. Biochem. Pharmacol..

[48] Sahay G, Alakhova DY, Kabanov AV (2010). Endocytosis of nanomedicines. J. Control. Release.

[49] Iversen TG, Skotland T, Sandvig K (2011). Endocytosis and intracellular transport of nanoparticles: Present knowledge and need for future studies. Nano Today.

[50] Kou L, Sun J, Zhai Y, He Z (2013). The endocytosis and intracellular fate of nanomedicines: Implication for rational design. Asian J. Pharm. Sci..

[51] Panyam J, Labhasetwar V (2003). Dynamics of endocytosis and exocytosis of poly(D, L-lactide-co-glycolide) nanoparticles in vascular smooth muscle cells. Pharm. Res..

[52] Hofmann D, Mailänder V (2013). Pharmacology of nanocarriers on the microscale: Importance of uptake mechanisms and intracellular trafficking for efficient drug delivery. Nanomedicine.

[53] Baltazar GC (2012). Acidic nanoparticles are trafficked to lysosomes and restore an acidic lysosomal pH and degradative function to compromised ARPE-19 cells. PLoS ONE.

[54] Mayle KM, Le AM, Kamei DT (2012). The intracellular trafficking pathway of transferrin. Biochim. Biophys. Acta - Gen. Subj..

[55] Stewart MP, Lorenz A, Dahlman J, Sahay G (2016). Challenges in carrier-mediated intracellular delivery: Moving beyond endosomal barriers. Wiley Interdiscip. Rev. Nanomed. Nanobiotechnol..

[56] Reix N (2012). In vitro uptake evaluation in Caco-2 cells and in vivo results in diabetic rats of insulin-loaded PLGA nanoparticles. Int. J. Pharm..

[57] Iturrioz-Rodríguez N, Correa-duarte MÁ, Valiente R, Mónica L, Fanarraga ML (2020). Engineering sub-cellular targeting strategies to enhance safe cytosolic silica particle dissolution in cells. Pharmaceutics.

[58] Furumoto K (2001). Biliary excretion of polystyrene microspheres depends on the type of receptor-mediated uptake in rat liver. Biochim. Biophys. Acta Gen. Subj..

[59] Zhang H (2018). Ligand size and conformation affect the behavior of nanoparticles coated with in vitro and in vivo protein corona. ACS Appl. Mater. Interfaces.

[60] Sahoo SK, Labhasetwar V (2005). Enhanced antiproliferative activity of. Mol. Pharm..

[61] Pisanti S, Picardi P, D’Alessandro A, Laezza C, Bifulco M (2013). The endocannabinoid signaling system in cancer. Trends Pharmacol. Sci..

[62] Qian ZM, Li H, Sun H, Ho K (2002). Targeted drug delivery via the transferrin receptor-mediated endocytosis pathway. Pharmacol. Rev..

[63] Li H, Qian ZM (2002). Transferrin/transferrin receptor-mediated drug delivery. Med. Res. Rev..

[64] Hansen SH, Sandvig K, Van Deurs B (1993). Clathrin and HA2 adaptors: Effects of potassium depletion, hypertonic medium, and cytosol acidification. J. Cell Biol..

[65] Chen CL (2009). Inhibitors of clathrin-dependent endocytosis enhance TGFβ signaling and responses. J. Cell Sci..

[66] Gao H (2013). Ligand modified nanoparticles increases cell uptake, alters endocytosis and elevates glioma distribution and internalization. Sci. Rep..

[67] Panyam J, Zhou W, Prabha S, Sahoo SK, Labhasetwar V (2002). Rapid endo-lysosomal escape of poly(DL-lactide- co glycolide) nanoparticles: implications for drug and gene delivery. FASEB J..

[68] Chang J (2009). Characterization of endocytosis of transferrin-coated PLGA nanoparticles by the blood-brain barrier. Int. J. Pharm..

[69] Durán-Lobato M, Carrillo-Conde B, Khairandish Y, Peppas NA (2014). Surface-modified P(HEMA-co-MAA) nanogel carriers for oral vaccine delivery: Design, characterization, and in vitro targeting evaluation. Biomacromol.

[70] dos Santos T, Varela J, Lynch I, Salvati A, Dawson KA (2011). Effects of transport inhibitors on the cellular uptake of carboxylated polystyrene nanoparticles in different cell lines. PLoS ONE.

[71] Salvati A (2013). Transferrin-functionalized nanoparticles lose their targeting capabilities when a biomolecule corona adsorbs on the surface. Nat. Nanotechnol..

[72] Berrecoso G, Crecente-Campo J, Alonso MJ (2020). Unveiling the pitfalls of the protein corona of polymeric drug nanocarriers. Drug Deliv. Transl. Res..

[73] Cózar-Bernal MJ (2011). Insulin-loaded PLGA microparticles: Flow focusing versus double emulsion/solvent evaporation. J. Microencapsul..

[74] Hu L, Mao Z, Zhang Y, Gao C (2011). Influences of size of silica particles on the cellular endocytosis, exocytosis and cell activity of HepG2 cells. J. Nanosci. Lett..

[75] Kitchens KM, Kolhatkar RB, Swaan PW, Ghandehari H (2008). Endocytosis inhibitors prevent poly(amidoamine) dendrimer internalization and permeability across caco-2 cells. Mol. Pharm..

[76] Zhang X (2014). In situ self-assembly of peptides in glucan particles for macrophage-targeted oral delivery. J. Mater. Chem. B.

[77] Guo M (2013). Mechanisms of chitosan-coated poly(lactic-co-glycolic acid) nanoparticles for improving oral absorption of 7-ethyl-10-hydroxycamptothecin. Nanotechnology.

[78] Snipstad S (2017). Labeling nanoparticles: Dye leakage and altered cellular uptake. Cytom. Part A.

