# Supplementary material for: Receptor-targeted nanoparticles modulate cannabinoid anticancer activity through delayed cell internalization
Source: Sci Rep. 2022 Jan 25;12:1297. doi: 10.1038/s41598-022-05301-z (PMC8789857; doi:10.1038/s41598-022-05301-z)
Supplement: Supplementary file 1 — Supplementary Information. [file 41598_2022_5301_MOESM1_ESM.pdf]

SUPPLEMENTARY INFORMATION

Receptor-targeted nanoparticles modulate cannabinoid anticancer activity through delayed cell internalization

Matilde Durán-Lobato[a*], Josefa Álvarez-Fuentes[a], Mercedes Fernández-Arévalo[a], Lucía Martín-Banderas[a]

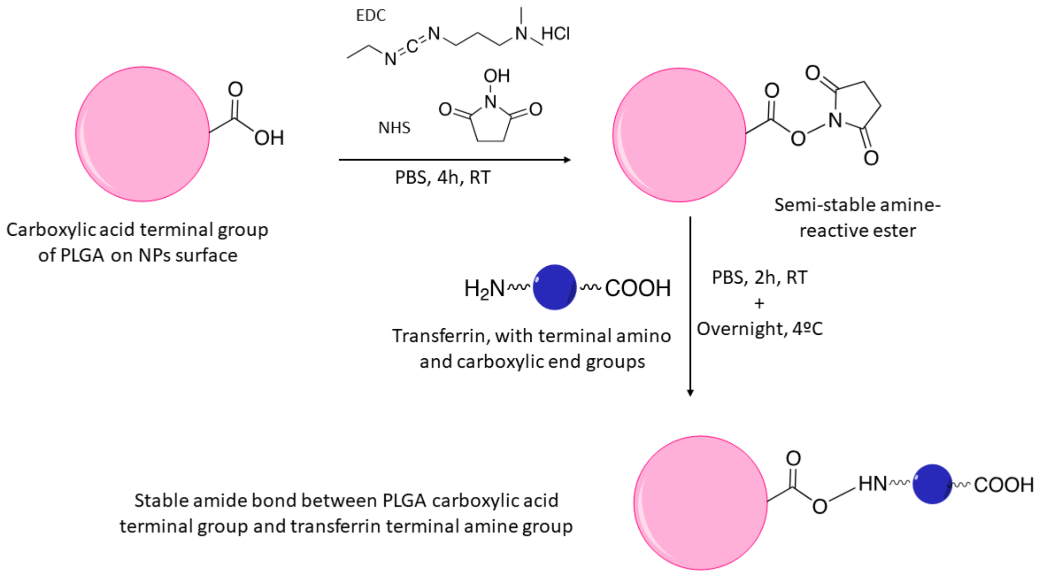

Figure S1. Schematic representation of transferrin conjugation to PLGA NPs through EDC/NHS chemistry.

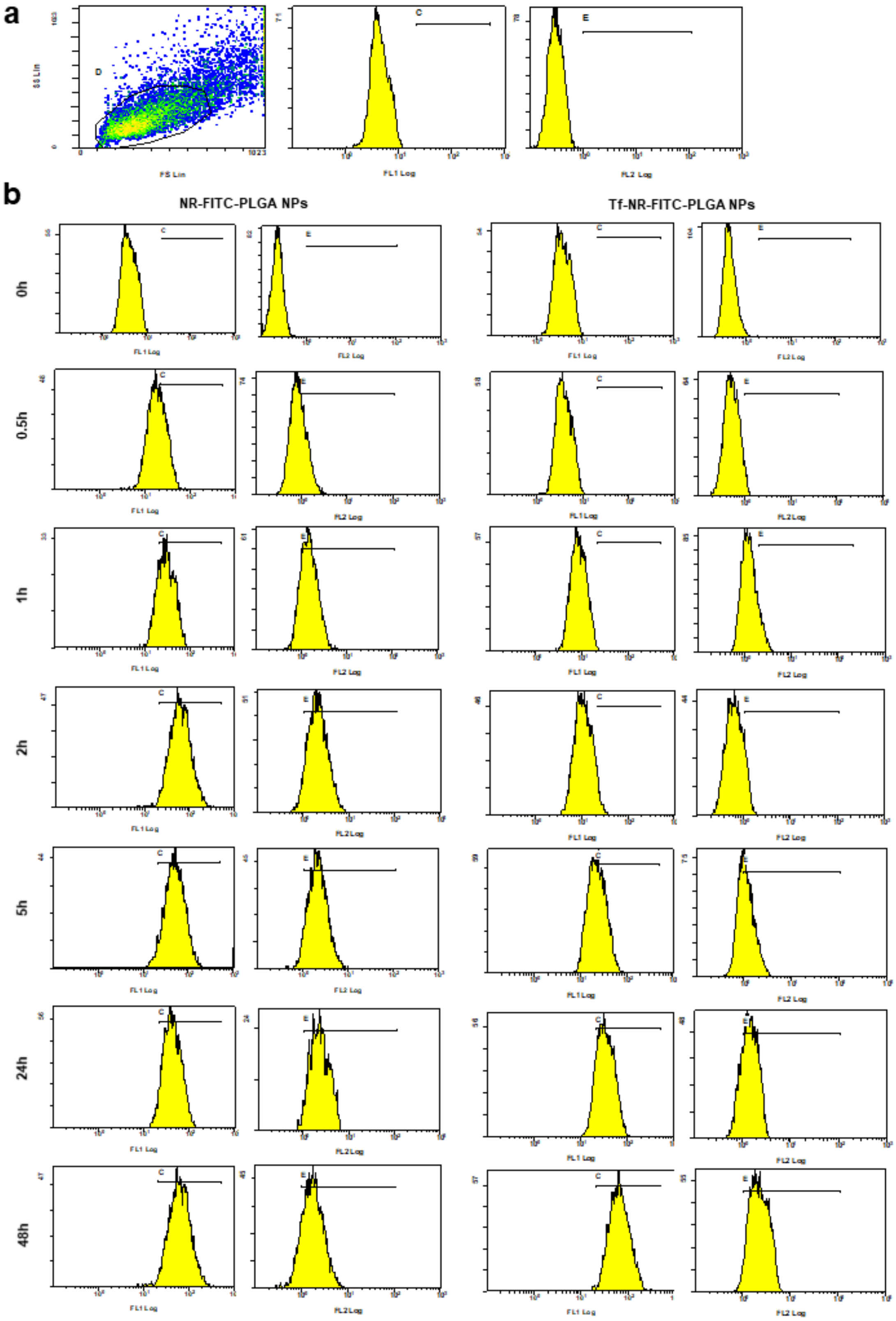

Figure S2. Flow cytometry histograms illustrating fluorescence from Caco-2 cells after incubation with the NPs. The histograms show one representative experiment comparing

plain NR-FITC-PLGA NPs vs. Tf-NR-FITC PLGA NPs in standard conditions (no inhibitors), corresponding to the complete set of data displayed in Figure 4a. (**a**) Setting of negative control: negative control for 0% fluorescence intensity was set by analyzing cells without NP treatment with a minimum of 10,000 cells counted per sample. A collection gate was subjectively chosen from the distribution in the side scatter (SS) vs. forward scatter (FS) dot plot to exclude cellular fragments and debris, and the obtained level of fluorescence was set as 0% in channels FL1 (corresponding to FITC signal) and FL2 (corresponding to Nile Red signal), yielding the regions for quantification of the normalized fluorescence intensity (C and E respectively). (**b**) Histograms displaying fluorescence from gated Caco-2 cells positive in channel FL1 (C region, corresponding to FITC signal) and channel FL2 (E region, corresponding to Nile Red signal) after incubation with NR-FITC-PLGA NPs vs. Tf-NR-FITC PLGA NPs at each time point assayed. Y-axis display number of counts, X-axis displays fluroescence intensity in channels FL1 (FITC) and FL2 (Nile Red).
